# Specific Detection and Identification of American Mulberry-Infecting and Italian Olive-Associated Strains of *Xylella fastidiosa* by Polymerase Chain Reaction

**DOI:** 10.1371/journal.pone.0129330

**Published:** 2015-06-10

**Authors:** Wei Guan, Jonathan Shao, Toufic Elbeaino, Robert E. Davis, Tingchang Zhao, Qi Huang

**Affiliations:** 1 Institute of Plant Protection, Chinese Academy of Agricultural Sciences, Beijing, China; 2 Floral and Nursery Plants Research Unit, Agricultural Research Service, United States Department of Agriculture, Beltsville, Maryland, United States of America; 3 Molecular Plant Pathology Laboratory, Agricultural Research Service, United States Department of Agriculture, Beltsville, Maryland, United States of America; 4 Istituto Agronomico Mediterraneo, Via Ceglie 9, 70010, Valenzano (BA), Italy; Oklahoma State University, UNITED STATES

## Abstract

*Xylella fastidiosa* causes bacterial leaf scorch in many landscape trees including elm, oak, sycamore and mulberry, but methods for specific identification of a particular tree host species-limited strain or differentiation of tree-specific strains are lacking. It is also unknown whether a particular landscape tree-infecting *X*. *fastidiosa* strain is capable of infecting multiple landscape tree species in an urban environment. We developed two PCR primers specific for mulberry-infecting strains of *X*. *fastidiosa* based on the nucleotide sequence of a unique open reading frame identified only in mulberry-infecting strains among all the North and South American strains of *X*. *fastidiosa* sequenced to date. PCR using the primers allowed for detection and identification of mulberry-infecting *X*. *fastidiosa* strains in cultures and in samples collected from naturally infected mulberry trees. In addition, no mixed infections with or non-specific detections of the mulberry-infecting strains of *X*. *fastidiosa* were found in naturally *X*. *fastidiosa*-infected oak, elm and sycamore trees growing in the same region where naturally infected mulberry trees were grown. This genotype-specific PCR assay will be valuable for disease diagnosis, studies of strain-specific infections in insects and plant hosts, and management of diseases caused by *X*. *fastidiosa*. Unexpectedly but interestingly, the unique open reading frame conserved in the mulberry-infecting strains in the U. S. was also identified in the recently sequenced olive-associated strain CoDiRO isolated in Italy. When the primer set was tested against naturally infected olive plant samples collected in Italy, it allowed for detection of olive-associated strains of *X*. *fastidiosa* in Italy. This PCR assay, therefore, will also be useful for detection and identification of the Italian group of *X*. *fastidiosa* strains to aid understanding of the occurrence, evolution and biology of this new group of *X*. *fastidiosa* strains.

## Introduction

*Xylella fasdiosa* inhabits plant xylem tissue and causes diseases in over 30 plant families. The diseases include agriculturally important Pierce’s disease of grapevine, almond leaf scorch, citrus variegated chlorosis, and coffee leaf scorch [1]. *X*. *fastidiosa* also causes bacterial leaf scorch (BLS) and decline in many landscape trees and shrubs including elm (*Ulmus* spp.), oak (*Quercus* spp.), sycamore (*Platanus* spp.), mulberry (*Morus* spp.), and oleander (*Nerium* oleander), and it is associated with BLS in many woody ornamentals such as maple (*Acer* spp.), box elder (*A*. *negundo)*, Japanese beech bonsai (*Fagus crenata)*, porcelain berry (*Ampelopsis brevipedunculata*) and crape myrtle (*Lagerstroemia indica*) [2–7].

*X*. *fastidiosa* is transmitted by xylem-feeding insects, such as sharpshooter leafhoppers (family Cicadellidae) and spittlebugs (family Cercopidae) [8,9]. It remains unknown, however, whether these insects can be colonized by and transmit more than one strain of *X*. *fastidiosa*, because adequate methods to specifically detect and identify strains of *X*. *fastidiosa* have not been available.

Mulberry is a low maintenance and attractive shade tree that can quickly mature into a stately ornamental tree that provides abundant berries for humans and wild life. Red mulberry (*Morus rubra* L.) is native to the eastern half of North America and is common in the United States. White mulberry (*Morus alba*) is native to Asia and was introduced in the United States along the Atlantic seaboard during colonial times in an effort to establish a silkworm industry [10]. It was also widely cultivated in Europe during the 18th and 19th centuries as a food source for silkworms.

BLS caused by *X*. *fastidiosa* in mulberry was first reported by Kostka et al. in 1986, when the disease was observed in red mulberry trees ranging from northern Virginia and Washington, D. C, along the east coast in Maryland, Delaware, eastern Pennsylvania, New Jersey, and north to New York State [11]. Subsequently, BLS has been reported on the white mulberry in Virginia [6], Washington, D.C. [12] and California [13], on both red and white mulberry in Nebraska [14], as well as on unspecified species of mulberry in Maryland [15] and Washington, D. C. [16]. A draft genome sequence of the mulberry-infecting strain Mul-MD, isolated from a mulberry tree growing in Maryland, and the complete genome sequence of the mulberry-infecting strain MUL0034, isolated from a white mulberry tree in California, have recently been determined [15,17].

Olive (*Olea europaea*) is another plant species that can be affected by *X*. *fastidiosa*. The plant is a long-living evergreen tree or shrub native to the Mediterranean, Asia and Africa. Its fruit is of major agricultural importance as a relish or as the source of olive oil. Olive trees can also be planted as beautiful ornamental trees [18]. *X*. *fastidiosa* had never been reported in Europe until 2013, when it was identified in aged olive trees affected by a disease named “Olive Quick Decline Syndrome (OQDS)”, as well as in leaf scorched almond and oleander trees growing near the diseased olive groves in southern Italy [19]. A draft genome sequence was determined in 2015 for CoDiRO, the new *X*. *fastidiosa* strain associated with OQDS in Italy [20], and three insects [*Philaenus spumarius*, *Neophilaenus campestris* and *Euscelis lineolatus* (a phloem feeder)] were reported as potential vectors of this Italian strain of *X*. *fastidiosa* [21,22].

Currently, *X*. *fastidiosa* is considered a single species containing four generally accepted subspecies. Subspecies *piercei*, *multiple*x and *sandyi* are found in the U. S. infecting mainly grapevines, tree species, and oleanders, respectively. Subspecies *pauca* is found in South America affecting citrus and coffee [23]. The recently discovered olive strains in Italy form a distinct cluster closely related to subspecies *pauca* [24,25]. Recently, Nunney et al. [17] proposed a new subspecies, *morus*, to include strains causing BLS in mulberry, although multiple sequence types are associated with this group of *X*. *fastidiosa* strains [17].

*X*. *fastidiosa* and its insect vectors occur in a wide variety of areas including where grape and landscape trees are growing. Little is known, however, about what strain(s) are present in wild or cultivated host plants growing near vineyards [26] or in landscape trees that test positive for *X*. *fastidiosa*, and whether more than one strain/subspecies of *X*. *fastidiosa* can co-exist in an individual plant of these different host species, especially if different host plant species are growing in close proximity [26,27]. In addition, in epidemiological studies, when insect vectors test positive for *X*. *fastidiosa*, it is critical to know what strain(s)/subspecies of *X*. *fastidiosa* that they carry. So far, although primers are available for PCR-based detection of *X*. *fastidiosa* at the species level [28–30], methodology for specific identification of a particular group of *X*. *fastidiosa* strains by a single pair of PCR primers has only been developed for citrus variegated chlorosis (CVC) strains [30] and for oleander-infecting strains [31]. A set of three primer pairs was developed [32] to differentiate grape-, almond- and oleander-infecting strains of *X*. *fastidiosa* [26,33].

In this communication, we describe the design and use of PCR primers that make it possible to specifically detect and identify both mulberry-infecting strains of *X*. *fastidiosa* in the U. S., and olive-associated strains in Italy.

## Material and Methods

### Bacterial strains and DNA extraction

Bacterial strains used in this study are listed in Table 1, and were grown as described [31]. Two strains of *X*. *fastidiosa*, Mul-KY and Maple-KY, were isolated in this study using the method of Huang and Sherald [6] from leaf petioles of *X*. *fastidiosa*-infected mulberry and maple tree samples, respectively, that were collected in Lexington, KY and kindly provided by N. Mundell and John Hartman (University of Kentucky, Lexington, KY). Genomic DNA from these strains was extracted using DNeasy tissue kit according to the manufacturer’s instructions (Qiagen Inc., Valencia, CA). A bacterial cell suspension was made by suspending a single colony of a *X*. *fastidiosa* strain in 50 μl of sterile water. The cell suspension was stored at -20°C for further use.

**Table 1:** Bacterial strains used in this study and results from PCRs using primer pair Mul-15040-F/R or 272-1-int/272-2-int.

				PCR product^a^	
Strain	Host	Origin	Reference or source	Mul-15040-F/R	272-1-int/272-2-int
*Xylella fastidiosa*					
Mul-MD	Mulberry	Maryland	[15]	+	+
Mul	Mulberry	Washington, DC	J. L. Sherald, Natl. Park Serv.	+	+
Mul-7	Mulberry	Nebraska	C. J. Chang, U. of Georgia	+	+
Mul-KY	Mulberry	Kentucky	This study	+	+
Sy	Sycamore	Washington, DC	J. L. Sherald, Natl. Park Serv.	-	+
Sy-Deer Creek 5	Sycamore	Georgia	C. J. Chang, U. of Georgia	-	+
Sy-VA	Sycamore	Virginia	[31,35]	-	+
Sy-VA2	Sycamore	Virginia	[31]	-	+
Oak	Oak	Washington, DC	J. L. Sherald, Natl. Park Serv.	-	+
Oak	Oak	Washington, DC	ATCC 35874	-	+
Oak 88–9	Oak	Florida	D. L. Hopkin, U. of Florida	-	+
Elm	Elm	Washington, DC	J. L. Sherald, Natl. Park Serv.	-	+
Maple-UCB	Maple	California	A. H. Purcell, UC-Berkeley	-	+
Maple-KY	Maple	Kentucky	This study	-	+
GA plum 2#4	Plum	Georgia	A. H. Purcell, UC-Berkeley	-	+
ALS1	Almond	California	[36]	-	+
Dixon	Almond	California	[36]	-	+
Temecula 1	Grape	California	J. S. Hartung, USDA-ARS	-	+
STL	Grape	California	[36]	-	+
UCLA	Grape	California	[36]	-	+
PCE-FG	Grape	Florida	C. J. Chang, U. of Georgia	-	+
PCE-RG	Grape	Florida	C. J. Chang, U. of Georgia	-	+
PD 95–2	Grape	Florida	D. L. Hopkin, U. of Florida	-	+
PD 95–8	Grape	Florida	D. L. Hopkin, U. of Florida	-	+
CVC	Citrus	Brazil	J. S. Hartung, USDA-ARS	-	+
Ann1	Oleander	California	[37]	-	+
T1B	Oleander	California	A. H. Purcell, UC-Berkeley	-	+
T5C	Oleander	California	A. H. Purcell, UC-Berkeley	-	+
TR2	Oleander	California	A. H. Purcell, UC-Berkeley	-	+
G	Oleander	Texas	[38]	-	+
Xf6	Olive	Gallipoli, Italy	[25]	+	+^b^
Xf9	Olive	Gallipoli, Italy	[25]	+	+ ^b^
*Enterobacter cloacae* 501 R3			J. S. Hartung, USDA-ARS	-	-
*Pseudomonas syringae* B1631			J. S. Hartung, USDA-ARS	-	-
*Xanthomonas axonopodis* pv. *citri* M1 1–4			J. S. Hartung, USDA-ARS	-	-
*Xanthomonas campestris* pv. *campestris* X6			J. S. Hartung, USDA-ARS	-	-

^a^ Presence or absence of the 312-bp PCR fragment amplified by Mul-15040-F/R, or the 472-bp band by 272-1-int/272-2-int primers [30] was indicated by “+” or “-”, respectively.

^b^Presence of the 733-bp PCR fragment amplified by *X*. *fastidiosa*-specific primers RST31/RST33 [29].

### Collection of and DNA extraction from plant samples

A total of forty-six plant DNA samples extracted from red oak (*Quercus rubr*a), pin oak (*Q*. *palustris*), elm (*Ulmus americana*), sycamore (*Platanus occidentalis*) and mulberry (*Morus alba*) growing in the Washington, D.C. area were kindly provided by Jordan Harris of the Yilmaz Balci lab (University of Maryland, College Park) [12].

A total of forty-six plant samples from 10 mulberry and 36 olive trees were collected from five different orchards located in *X*. *fastidiosa*-affected area of Gallipoli in Lecce province, in the south of Italy. Tissues used for bacterial detection consisted of leaf petioles and mid-veins excised from mature leaves, from which total DNA was extracted using Qiagen’s DNeasy Plant Mini kit, according to the manufacturer’s instructions.

### Identification of unique DNA sequences and design of PCR primers

Four complete genomes of *X*. *fastidiosa* strains, Temecula 1 (AE009442) from Pierce’s-diseased grapevine, 9a5c (AE003849) from variegated chlorosis-diseased citrus, and M12 (CP000941) and M23 (CP001011) from leaf scorch-diseased almond, as well as the draft genome of the mulberry-infecting strain Mul-MD (AXDP00000000) [15], were downloaded from NCBI’s whole genome and shotgun reads database. Proteins encoded by predicted genes of the five *X*. *fastidiosa* strains were compared using a bi-directional BLAST between amino acid sequences from the five strains. The e-value threshold was e^-04^ or less for two proteins to be considered a match. A protein was considered strain specific if BLAST yielded no hits or all hits had an e-value greater than e^-04^. Mul-MD strain-unique genes were then compared with the genome of the other mulberry strain MUL0034 (CP006740.1), and with other complete and draft genomes of *X*. *fastidiosa* strains in GenBank, to ensure their uniqueness. Primers were designed based on a unique gene selected using the criteria and software as described previously [34]. A search for nucleotide sequence matches of the primers and amplicons was conducted using BLASTn to query the nucleotide collection (nr/nt) and whole-genome shotgun contigs (wgs) databases in GenBank.

### PCR assay conditions

PCR using the specific primers we designed in this study was performed in a 20-μl volume containing 5 pmol of each primer, and approximately 20 ng of bacterial genomic DNA, 1 μl of bacterial cell suspension or 2 μl of plant DNA extract in 1 x GoTaq Green Master Mix (Promega, Madison, WI). The PCR conditions were 1 cycle of 5 min at 95°C, 30 cycles of 1 min at 95°C, 1 min at 62°C, and 1 min at 72°C, with a final extension of 10 min at 72°C. For comparison, PCR was also performed with the previously reported *X*. *fastidiosa*-specific primer pair 272-1-int and 272-2-int [30] or primer pair RST31 and RST 33 [29], except that an annealing temperature of 64°C was used for the 272-1-int and 272-2-int primers. The PCR products were visualized as described [34].

### Purification, Cloning and Sequencing of PCR Products

Selected PCR products were purified from agarose gels with the QIAquick gel extraction kit (Qiagen Inc.). The purified DNA fragment was cloned into the TOPO vector using the TOPO TA cloning kit (Invitrogen Corporation, Carlsbad, CA, USA) according to the manufacturer’s instructions. Desired clones were purified using Qiagen’s QIAprep spin miniprep kit. Both strands of the inserts in the clones were sequenced commercially using M13 forward and reverse primers, respectively.

## Results

By comparing the open reading frames (ORFs) of *X*. *fastidiosa* mulberry-infecting strain Mul-MD with those in the complete genomes of *X*. *fastidiosa* strains CVC 9a5c, Temecula 1, M12 and M23, we identified 110 unique ORFs present in Mul-MD (unpublished results). Upon further comparison to the ORFs of other complete or draft genomes of *X*. *fastidiosa* strains in GenBank using BLASTp, one of these, ORF EWG15040.1 was also found in mulberry-infecting strain MUL0034 and matched 100% at the amino acid level with the ORF AIC14133.1, as well as in the recently sequenced olive-infecting strain CoDiRO isolated in Italy, sharing 98% amino acid sequence identity with the ORF KIA57852.1. This ORF was not found in any other genome-sequenced *X*. *fastidiosa* strains and was annotated as encoding a hypothetical protein. The protein contains a putative conserved thioredoxin reductase domain, similar to a putative bacillithiol system oxidoreductase in the YpdA family. Since this ORF was not found in any of the North and South American non-mulberry-infecting *X*. *fastidiosa* strains, its nucleotide sequence was used to design a primer set containing the forward primer Mul-15040-F and the reverse primer Mul-15040-R (Table 2).

**Table 2:** List of primers designed in this study, size of PCR product, target gene, and specificity of primers.

Primer pair	Sequences (5’– 3’)	Size of PCR product (bp)	Target	Specificity^a^
Mul-15040-F	ATTTTCGCGATTTTGGAGTT	312	Hypothetical protein containing a region of putative bacillithiol system oxidoreductase,YpdA family	American mulberry-infecting and Italian olive-associated strains of *X*. *fastidiosa*
Mul-15040-R	TTCTTGTGTACTCCGCCTCA			

^a^Primers amplified product only from tested mulberry and olive strains of *X*. *fastidiosa*.

**Fig 1:**
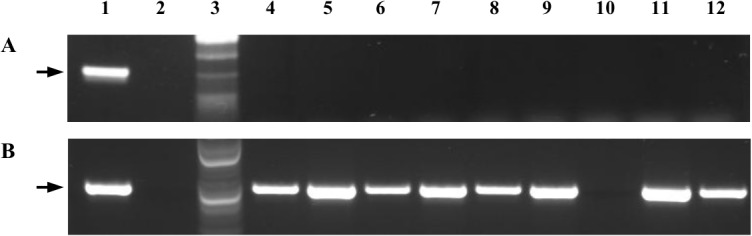
Amplification of *Xylella fastidiosa* DNA from extracted genomic DNA templates or whole cells added to reaction mixtures. PCRs were primed by primer pairs Mul-15054-F/R (A) or 272-1-int/272-2-int (B). Lanes: 1, Mul-MD; 2, no-template water control; 3, 100-bp DNA ladder; 4, Elm; 5, Oak-ATCC 35874; 6, Maple-KY; 7, ALS1; 8, Temecula 1; 9, CVC; 11, Sy; 12, Dixon. Note that the 312-bp DNA product (arrow in A) were amplified only from mulberry-infecting strain Mul-MD in PCR with the Mul-15054-F/R primers, but the 472-bp DNA product (arrow in B) was amplified from all *X*. *fastidiosa* strains in PCRs with the 272-1-int/272-2-int primers. No PCR product was observed in PCRs containing template DNA from the out-group bacterium *Xanthomonas Campestris* pv. *campestri*s X6 (lane 10).

The specificity of the primer set was assessed by PCR using template DNA extracted from cultured strains, or aliquots of suspensions of whole cultured cells. These PCRs involved 30 *X*. *fastidiosa* strains including four targeted mulberry-infecting strains and 26 non-target strains originally isolated and cultured from sycamore, oak, elm, maple, plum, almond, grape, citrus, and oleander in different geographic locations from north and south of America (Table 1). Four other plant-associated bacteria, *Enterobacter cloacae*, *Pseudomonas syringae*, *Xanthomonas axonopdis* pv. *citri* and *X*. *campestris* pv. *campestris*, were included as out-groups for the comparison (Table 1). The primer set Mul-15040-F/R primed amplification of a 312-bp product from all four mulberry-infecting strains that were originally isolated from mulberry trees growing in Maryland, Washington, D.C., Kentucky and Nebraska, respectively (Table 1, Fig 1A). No PCR product was observed when any of the other 26 *X*. *fastidiosa* strains, or any of the four out-group bacterial strains, served as PCR template (Table 1, Fig 1A). On the other hand, PCR primed by *X*. *fastidiosa*-universal primer pair 271-1-int/272-2-int yielded a 472-bp product from all 30 strains of *X*. *fastidiosa*, but from none of the four out-group bacteria (Table 1, Fig 1B).

The 312 bp mulberry-infecting strain specific product, amplified from the genomic DNA of strain Mul-MD in PCRs primed by primer pair Mul-15040-F/R, shared 100% nucleotide sequence identity with nucleotides 3766 to 4077 in contig AXDP01000003 of mulberry-infecting strain Mul-MD and with nucleotides 2,431,341 to 2,431,652 in the genome of mulberry-infecting strain MUL0034 of *X*. *fastidiosa*. It also shared 98% nucleotide sequence identity with nucleotide 257,289 to 256,978 in contig JUJW01000007 of the Italian olive strain CoDiRO. No significant nucleotide sequence identity was found with any other genomes of *X*. *fastidiosa* strains or other species in GenBank.

The primer pair Mul-15040-F/R was also tested against DNA samples extracted from 46 landscape trees (seven mulberry, eight sycamore, six elm, ten pin oak and 15 red oak) growing in different urban areas in Washington D. C. (Table 3). The 46 DNA samples, extracted from BLS symptomatic or asymptomatic tree samples, were first tested by PCR using *X*. *fastidiosa*-universal primers 272-1-int and 272-2-int to determine whether they contain *X*. *fastidiosa*. All but two mulberry samples tested positive for *X*. *fastidiosa* (Table 3); these two mulberry samples were therefore used as negative controls in further tests. PCR using the primer set Mul-15040-F/R primed amplification of a 312-bp DNA fragments from all five *X*. *fastidiosa*-positive mulberry tree samples (Table 3). No PCR product was observed from the two *X*. *fastidiosa*-negative mulberry samples, nor from any of the other 39 oak, elm and sycamore tree samples (Table 3).

**Table 3:** Plant samples used in this study and a comparison of associated PCR results using specific primer set Mul-15040-F/R developed in this study vs. *Xylella fastidiosa*-universal primers.

Country	Plant	# of plants	# of PCR (+) using	
			*X*. *fastidiosa*-specific primers^a^	Mul-15040-F/R
U. S.	Mulberry	7	5	5
	Sycamore	8	8	0
	Elm	6	6	0
	Pin oak	10	10	0
	Red Oak	15	15	0
Italy	Olive	36	36	36
	Mulberry	10	0	0

^a^Plant samples from the U. S. were tested by the primer pair 272-1-int and 272-2-int [30], and those from Italy by RST31 and RST33 [29].

**Fig 2:**
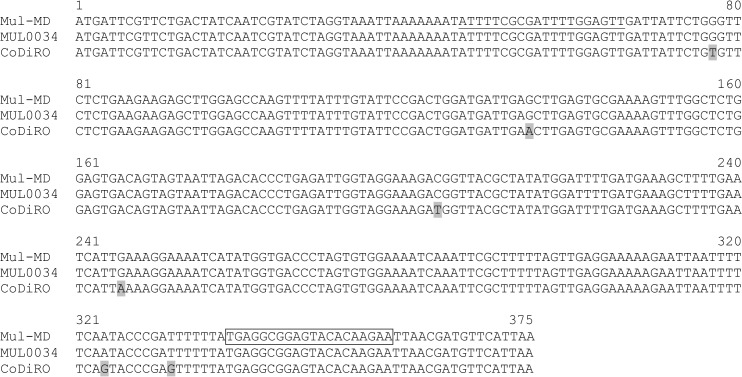
Alignment of nucleotide sequences of the open reading frame conserved among the two mulberry-infecting strains Mul-MD and MUL0034, as well the Italian olive-associated strain CoDiRO of *X*. *fastidiosa*. The forward primer Mul-15040-F is underlined and the reverse primer Mul-15040-R (reversed and complementary) is boxed in the Mul-MD strain. The nucleotides in CoDiRO that are different from the Mul-MD and MUL0034 strains are shaded in gray. Numbers indicate the size of the nucleotide sequence.

Since the primer pair Mul-15040-F/R matched 100% with annealing sites in DNA of the Italian olive strain CoDiRO (Fig 2), the primer pair was also tested against DNA samples extracted from 36 symptomatic olive tree samples collected from Gallipoli in Lecce province, southern Italy. As a control, the primer pair was also tested on DNA extracted from two cultured strains of *X*. *fastidiosa*, Xf6 and Xf9, isolated from diseased olive trees in Italy [25]. PCRs primed by Mul-15040-F/R amplified a 312-bp DNA fragment from both of the cultured Italian olive strains (Table 1), as well as from all the 36 olive tree samples (Table 3). The PCR-amplified DNA fragments from two of the 36 tree samples were randomly chosen for cloning and sequencing. The sequences of both clones were 312-bp in length and identical to nucleotides 257,289 to 256,978 in contig JUJW01000007 of the Italian olive-associated strain CoDiRO. The presence of *X*. *fastidiosa* in the 36 olive tree samples was also confirmed by PCR using *X*. *fastidiosa* species-specific primer pair RST31/RST33 (Table 3). When mulberry trees near the five affected olive orchards were surveyed, no leaf scorch symptoms were observed. When DNA samples extracted from ten asymptomatic mulberry tree samples were tested using primer pair Mul-15040-F/R, no PCR product was observed from any of the ten samples (Table 3). Similarly, PCR product was not observed when the ten asymptomatic mulberry samples were tested using *X*. *fastidiosa* universal primers RST31 and RST33, confirming the absence of *X*. *fastidiosa* in those Italian mulberry samples (Table 3).

## Discussion

In this communication we describe a PCR assay for specific detection and identification of *X*. *fastidiosa* strains that cause BLS in mulberry trees in the U. S. and those that occur in association with the OQDS disease in Italy. Initially, the specific PCR primers were designed based on a unique ORF that occurred only in the two published genomes of mulberry-infecting *X*. *fastidiosa* strains, not in any other North or South American *X*. *fastidiosa* strains sequenced so far. We designed primers Mul-15040-F/R that prime amplification of DNA fragments only from mulberry–infecting strains that were isolated from different geographic locations, even though DNA from all tested strains of *X*. *fastidiosa* was amplified by *X*. *fastidiosa*-universal primer pair 272-1-int/272-2-int. These results demonstrated the specificity of the primer pair for mulberry-infecting strains of *X*. *fastidiosa*. When the nucleotide sequence of the Mul-15040-F/R PCR amplicon was used as query to search the GenBank database, we found that although sharing a 98% sequence identity with the Italian olive strain CoDiRO, it shared 100% sequence identity only with a 312-bp region of the two sequenced mulberry strains MUL-MD and MUL0034, not with any other sequenced strains of *X*. *fastidiosa* in GenBank, further confirming the specificity of the amplicon among the North and South American *X*. *fastidiosa* strains. When tested in PCRs containing DNA templates extracted from infected plants, the primer set successfully identified the five *X*. *fastidiosa*-infected mulberry samples, out of the 44 tree samples naturally infected with different strains of *X*. *fastidiosa*, confirming their usefulness in detecting target *X*. *fastidiosa* strains in plants and also suggesting the absence of the mulberry-infecting strains in *X*. *fastidiosa*-infected oak, elm and sycamore trees even though those trees were from the same geographic location where *X*. *fastidiosa*-infected mulberry trees were located.

Based on our experience, mulberry-infecting *X*. *fastidiosa* strains grow more rapidly *in vitro* than strains isolated from other landscape trees, taking about 7 days to produce visible colonies on selective medium plates. Their growth rates are similar to those of the Pierce’s disease strains, although they are genetically different from the Pierce’s disease and other *X*. *fastidiosa* strains, and they are non-pathogenetic on grapevine and oleander [13]. Within the group of mulberry strains of *X*. *fastidiosa*, different genotypes have been reported among *in vitro* cultured strains [17] and among strains detected in field-collected tissue samples from naturally infected mulberry plants [12]. Our primer set Mul-15040-F/R, however, permitted detection of all the cultured mulberry strains in our collection, as well as those strains present in naturally infected mulberry, suggesting the robustness of our primers.

For over 25 years, *X*. *fastidiosa* has been found to cause or be associated with BLS in landscape trees, but knowledge about the etiology and epidemiology of *X*. *fastidiosa*-associated BLS has remained limited. When an insect vector or a new plant species is found harboring *X*. *fastidiosa* in places where diverse *X*. *fastidiosa*-infected agricultural crops, such as grapevine or/and landscape tree species including mulberry, are present or in close proximity, it is critical to determine the identity/genotype of the particular strain(s) that is/are carried by the vector or present in the plant species, in order to develop an effective control strategy. The specific detection and identification method developed in the present study will help to address these questions, and improve understanding of relationships among insect vectors, *X*. *fastidiosa* strain genotypes, and their hosts, as well as aiding the design of management strategies to control important *X*. *fastidiosa*-induced plant diseases.

One unexpected but interesting finding of this study is the discovery that a unique ORF, encoding a hypothetical protein and conserved in mulberry-infecting strains of *X*. *fastidiosa*, is also found in the recently sequenced genome of the olive strain CoDiRO isolated in Italy, but not in any other *X*. *fastidiosa* strains sequenced so far. *X*. *fastidiosa* has been found in olive in California before [3,18], but the olive strains from California are very different from those currently known in Italy. Phylogenetic analysis revealed that the California olive strains belong to the subspecies *multiplex* [3,18]. An alignment of concatenated sequences of *pilU* and seven housekeeping genes—*cysG*, *gltT*, *holC*, *malF*, *leuA*, *nuoL*, and *petC* revealed that the California olive strains share 99.97 to 100% nucleotide sequence identity of these genes with the subspecies *multiplex* strain M12 [18]. In contrast, the Italian olive-associated strains are more closely related to the subspecies *pauca* and form a distinct cluster that is different from the citrus and coffee-infecting *X*. *fastidiosa* strains, but in the same clade as the identified *pauca* strains [24,25]. Since we do not have the California olive strains, we could not test whether our primer pair can amplify these strains. It seems very unlikely, however, that DNA from the olive strains from California would be amplified by our primers, since the PCR target sequences are not present in the sequenced subspecies *multiplex* strains M12 and Dixon, which are most closely related to the olive strains in California. Moreover, no amplicon was detected in PCRs primed by our primers from template DNA of strain Dixon or other strains in subspecies *multiplex*, including sycamore-, oak-, elm-, maple- and plum-infecting strains (Table 1). On the contrary, however, our primers Mul-1540-F/R should amplify DNA of the olive strains from Italy, since the sequences of the primer pair match 100% with those of the olive-associated strain CoDiRO from Italy with the same amplicon sizes (Fig 2). As predicted, when tested against 2 cultured Italian olive strains and 36 field collected olive tree samples in Italy, the primer pair detected *X*. *fastidiosa* in both of the tested cultures (Table 1) and all of the olive tree samples collected from affected olive groves (Table 3). It does not seem that mulberry trees near the infected olive groves in Italy have been affected by *X*. *fastidiosa*, since PCRs using both primer pairs Mul-1540-F/R and RST31/RST33 yielded no product. Future research is needed to monitor mulberry trees during different growing seasons. Our study suggests that the specific primers we developed will be useful not only for specific detection and differentiation of mulberry-infecting strains in the U. S., but also for specific detection of the newly discovered olive-associated strains in Italy, as well as to differentiate the olive-associated strains from other strains of *X*. *fastidiosa*, if any are found in Italy or Europe in the future. Future research is needed to learn why this particular ORF is conserved only in *X*. *fastidiosa* strains affecting mulberry trees in the U. S. and olive trees in Italy, how this ORF evolved and what biological advantages this ORF might offer to those strains. It would be wise to search for other plant species in Italy near the infected olive groves to determine whether they are infected by *X*. *fastidiosa* and, if so, by which strains of *X*. *fastidiosa*. Finally the roles that insect vectors, including the spittlebugs identified in Italy, and host plant species such as mulberry and olive play in the conservation of this ORF are worthy of study.
